# Supplementary material for: Seminoma and Embryonal Carcinoma Footprints Identified by Analysis of Integrated Genome-Wide Epigenetic and Expression Profiles of Germ Cell Cancer Cell Lines
Source: PLoS One. 2014 Jun 2;9(6):e98330. doi: 10.1371/journal.pone.0098330 (PMC4041891; doi:10.1371/journal.pone.0098330)
Supplement: File S1 — ZIP file containing DMRforPairs output for significant regions. Please start from the html files. (ZIP) [file pone.0098330.s008.zip › figures/33755.pdf]

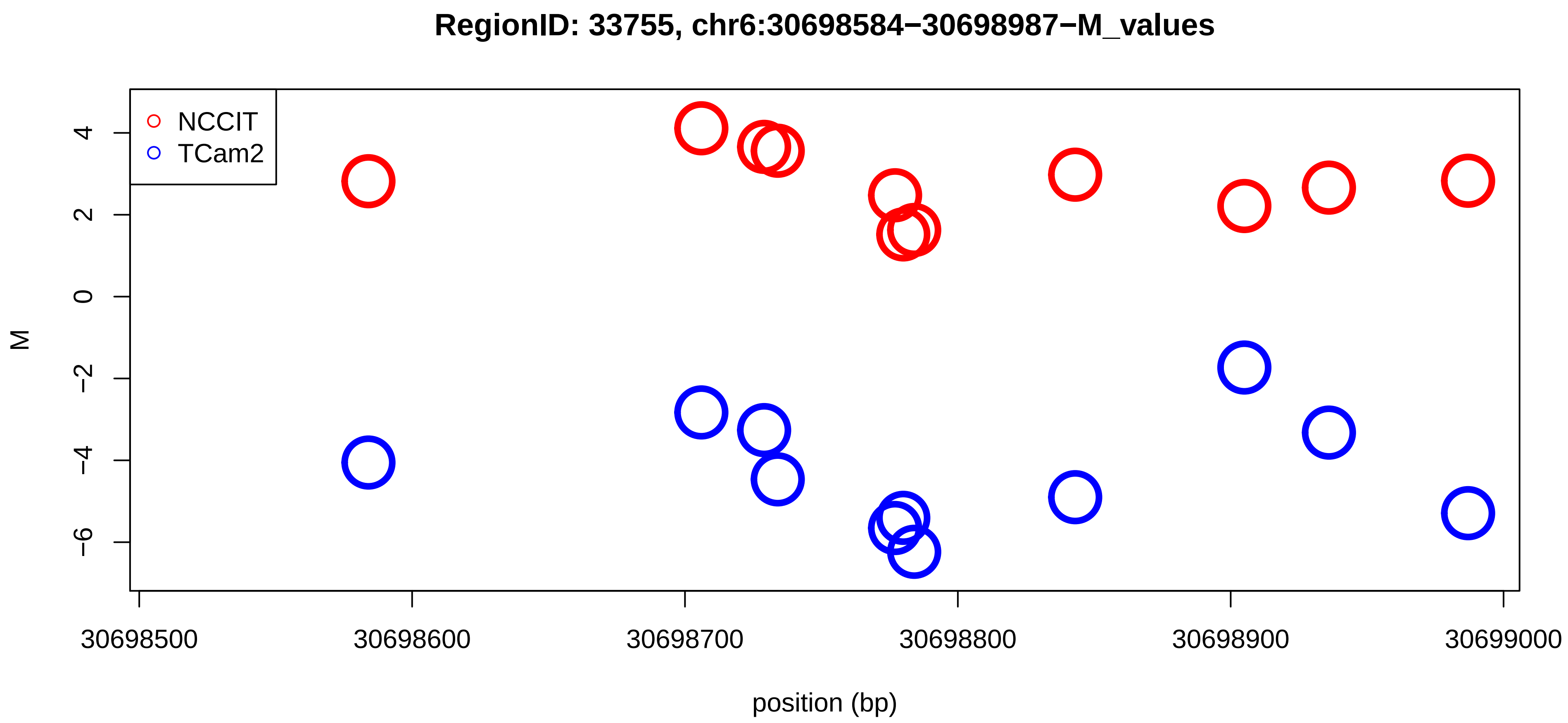
RegionID: 33755, chr6:30698584−30698987−M_values
NCCIT
TCam2
M
4
2
0
−2
−4
−6
30698500
30698600
30698700
30698800
30698900
30699000
position (bp)

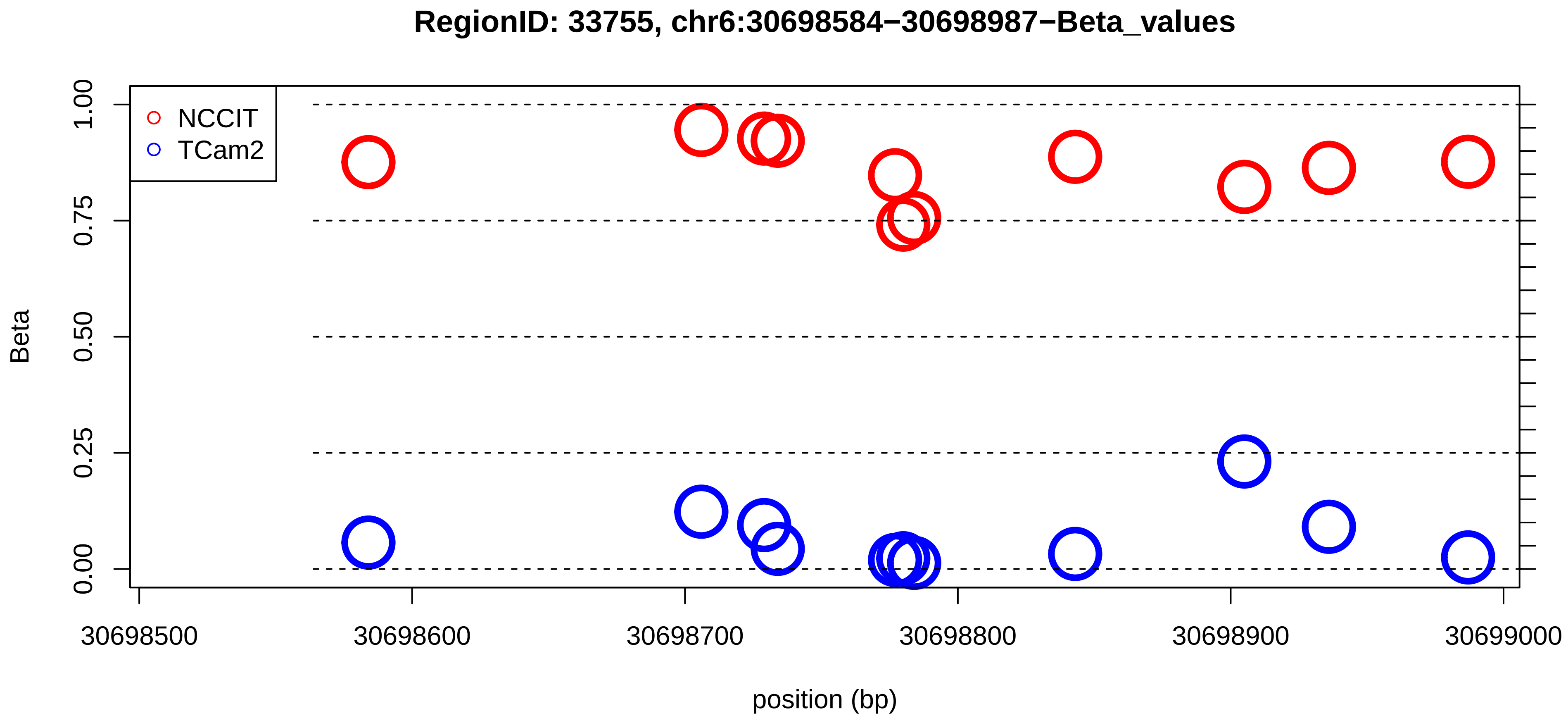
RegionID: 33755, chr6:30698584−30698987−Beta_values
NCCIT
TCam2
Beta
1.00
0.75
0.50
0.25
0.00
30698500
30698600
30698700
30698800
30698900
30699000
position (bp)

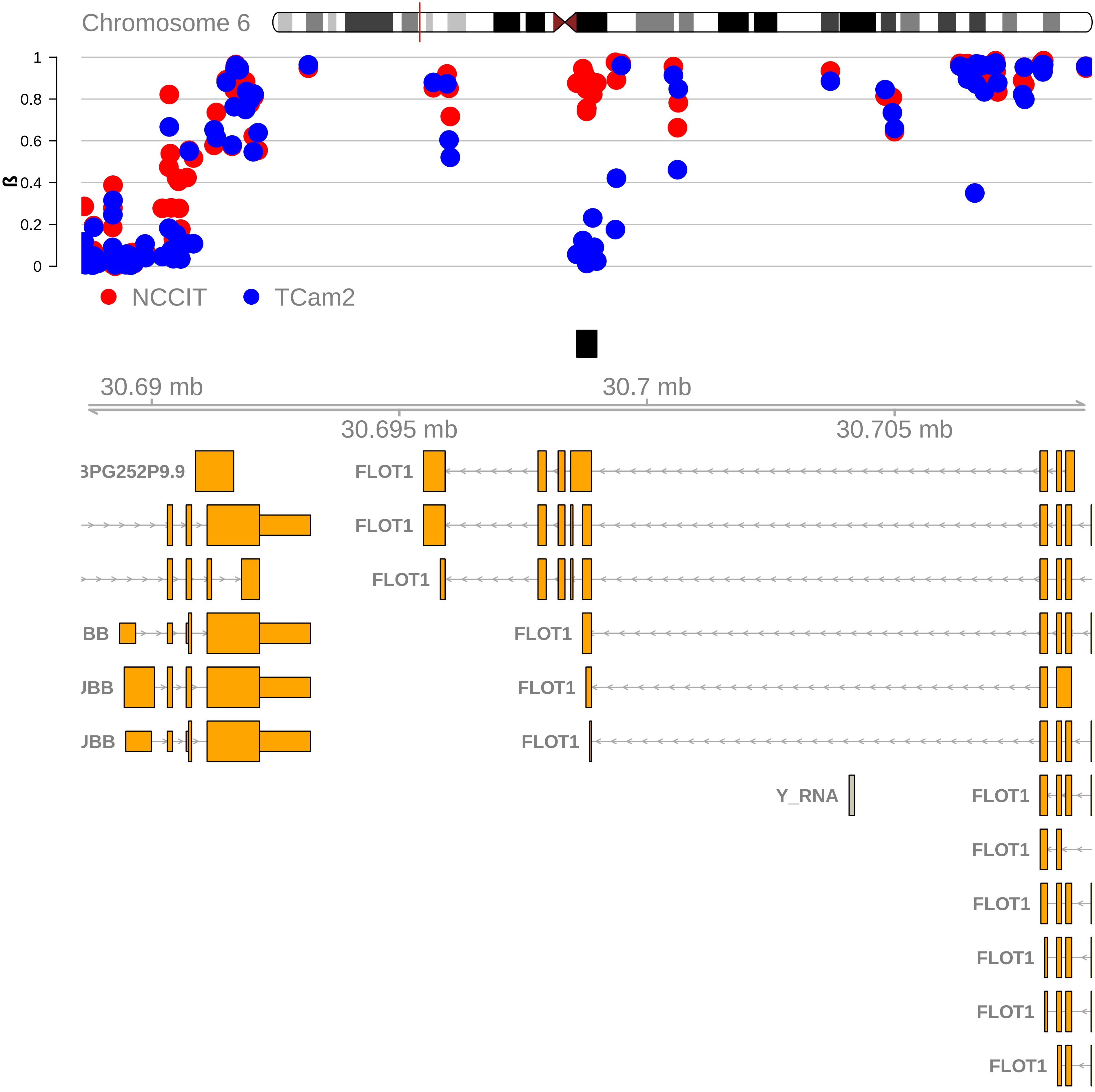

Chromosome 6
ß
1
0.8
0.6
0.4
0.2
0
NCCIT
TCam2
30.69 mb
30.7 mb
30.695 mb
30.705 mb
FLOT1
Y_RNA
